# Platelet-rich plasma alleviates neuropathic pain in osteoarthritis by downregulating microglial activation

**DOI:** 10.1186/s12891-024-07437-7

**Published:** 2024-05-09

**Authors:** Xiao Yan, Yinshuang Ye, Lin Wang, Junqiang Xue, Nana Shen, Tieshan Li

**Affiliations:** Department of Rehabilitation Medicine, The Affiliated Hospital of Qingdao University, Qingdao, Shandong Province People’s Republic of China

**Keywords:** Neuropathic pain, Platelet-rich plasma, Knee osteoarthritis, Microglial activation, Nerve injury

## Abstract

**Background:**

The development of neuropathic pain (NP) is one of the reasons why the pain is difficult to treat, and microglial activation plays an important role in NP. Recently, platelet-rich plasma (PRP) has emerged as a novel therapeutic method for knee osteoarthritis (KOA). However, it’s unclarified whether PRP has analgesic effects on NP induced by KOA and the underlying mechanisms unknown.

**Purpose:**

To observe the analgesic effects of PRP on NP induced by KOA and explore the potential mechanisms of PRP in alleviating NP.

**Methods:**

KOA was induced in male rats with intra-articular injections of monosodium iodoacetate (MIA) on day 0. The rats received PRP or NS (normal saline) treatment at days 15, 17, and 19 after modeling. The Von Frey and Hargreaves tests were applied to assess the pain-related behaviors at different time points. After euthanizing the rats with deep anesthesia at days 28 and 42, the corresponding tissues were taken for subsequent experiments. The expression of activating transcription factor 3 (ATF3) in dorsal root ganglia (DRG) and ionized-calcium-binding adapter molecule-1(Iba-1) in the spinal dorsal horn (SDH) was detected by immunohistochemical staining. In addition, the knee histological assessment was performed by hematoxylin-eosin (HE) staining.

**Results:**

The results indicated that injection of MIA induced mechanical allodynia and thermal hyperalgesia, which could be reversed by PRP treatment. PRP downregulated the expression of ATF3 within the DRG and Iba-1 within the SDH. Furthermore, an inhibitory effect on cartilage degeneration was observed in the MIA + PRP group only on day 28.

**Conclusion:**

These results indicate that PRP intra-articular injection therapy may be a potential therapeutic agent for relieving NP induced by KOA. This effect could be attributed to downregulation of microglial activation and reduction in nerve injury.

## Introduction

Knee osteoarthritis (KOA) is a common chronic degenerative disease characterized by articular cartilage degeneration, synovitis, subchondral bone remodeling, and joint muscle atrophy. KOA has the highest prevalence among all types of arthritis and affects approximately 15% of the world’s population [1]. The prominent symptom of KOA is pain, which is the main cause of disease-related disability [2, 3].Currently, treatment methods that can impede the progression of osteoarthritis joint injury are limited [4]and they can only provide temporary relief of symptoms, inevitably leading to complications and adverse reactions [5]. Consequently, finding a more effective and safer treatment for KOA with neuropathic pain (NP) remains a top priority, both in scientific research and clinical practice.

Traditionally, KOA pain is caused by the stimulation and activation of nociceptive receptors after tissue injury or inflammation inside and outside the joint. However, according to current clinical evidences, 34% of KOA patients have NP manifestations such as resting pain, burning pain, electric shock sensation and numbness [6]. The pain intensity of many patients is inconsistent with radiographic evidence [7, 8]. These findings all suggest that the pain of KOA patients is not only caused by local joint structure damage, but also may be related to injury of peripheral sensory nerve innervating joint and alteration of central nervous circuit [9]. Previous studies have confirmed that after KOA joint injury, the release of inflammatory cytokines in the early stage initiates inflammatory pain [10, 11], and then with the exposure of subchondral bone, abundant sensory nerve endings in it will be damaged due to exposure [12]. Then the expression of activating transcription factor 3 (ATF3), a specific marker for nerve injury, is up-regulated in the dorsal root ganglia (DRG), and subsequently the expression of adenosine triphosphate (ATP) in the spinal cord is significantly increased [13]. P2 × 4 and P2 × 7 receptors promote the activation of spinal microglia [14, 15], and microglia activation plays an important role in the occurrence and maintenance of chronic NP [16, 17]. After microglia activation, pro-inflammatory cytokines are further up-regulated [18], leading to further expansion of neuroinflammation and aggravation of NP. Therefore, peripheral nerve injury innervating the joint is a key mechanism leading to NP induced by KOA, and reducing nerve damage may become a new path to treat chronic NP in KOA.

Platelet-rich plasma (PRP) is an autologous serum that contains concentrated platelets, several growth factors, and cytokines. PRP has the advantages of having preferable therapeutic effects, autologous sources, and better safety, and is widely used in sports trauma [19]. Furthermore, preliminary investigations have demonstrated that PRP possesses the capacity to stimulate neurotrophic factor synthesis and significantly enhance Schwann cell migration [20, 21], thereby ameliorating peripheral nerve injury and alleviating NP. The effects of PRP have been investigated in several pain syndromes, including diabetic neuralgia and carpal tunnel syndrome [22]. Additionally, evidences showed that injection of PRP subcutaneously into burn scars in rats could lead to significant reduction of NP [20]. Nonetheless, previous studies on the analgesic efficacy of PRP in KOA mainly focused on early-stage inflammatory pain [21–23].The analgesic effect of PRP on NP induced by KOA has been scarcely investigated and remains unclear.

As far as we know, there have been no reports on the effects and underlying mechanisms of PRP on NP induced by KOA. Our study firstly explores the mechanisms through which PRP alleviates NP induced by KOA, with a specific focus on the downregulation of microglial activation. This might contribute to a better understanding of the pathophysiology in NP induced by KOA and further expand the clinical application of PRP in pain management.

## Materials and methods

### Animals

Adult male Sprague–Dawley (SD) rats weighing 180–220 g were provided by the Experimental Animal Center of Qingdao University (Qingdao, China). They were housed under alternating light-dark (12 h light/12 h dark) cycles, and the room temperature was maintained at 23 ± 1 °C with free access to food and water. All experimental protocols were approved by the Animal Care and Use Committee of Qingdao University and were carried out in accordance with the guidelines of the International Association for the Study of Pain [24].

### PRP preparation

Whole blood from four male SD rats were drawn preoperatively via abdominal aortic puncture into tubes containing 3.8% sodium citrate. PRP was obtained from anticoagulated blood after centrifugation at 800 rpm for 15 min at 25 °C. The platelets in whole blood and PRP were counted automatically using a hematology analyzer. The concentration of platelets achieved in PRP was 3–4 times higher than at baseline. PRP was activated by freezing at − 80 °C for 24 h and incubated at 37 °C for 1 h. After incubation, the activated PRPs were centrifuged at 12,000 × g for 2 min to separate the debris. The supernatant was collected and stored at − 80 ℃ until further use [25].

### Induction of arthritis and treatment

In this study, 30 rats were randomly divided into three groups based on the randomization table, namely monosodium iodoacetate (MIA) + PRP group, MIA + normal saline (NS) group and sham group, with 10 rats in each group. For rats in MIA + PRP group and MIA + NS group, 60 µl 80 mg/mL MIA (Sigma, I2512) into their left knee joints to induce joint degeneration [26]. The appearance of touch-induced pain and hyperalgesia is regarded as successful modeling. In addition, rats in the sham group were selected to inject equal volume NS into their left knee joints. Paw withdrawal threshold (PWT) and paw withdrawal latency (PWL) were measured at 1, 3, 7and 14 days after modeling.

**Fig. 1:**
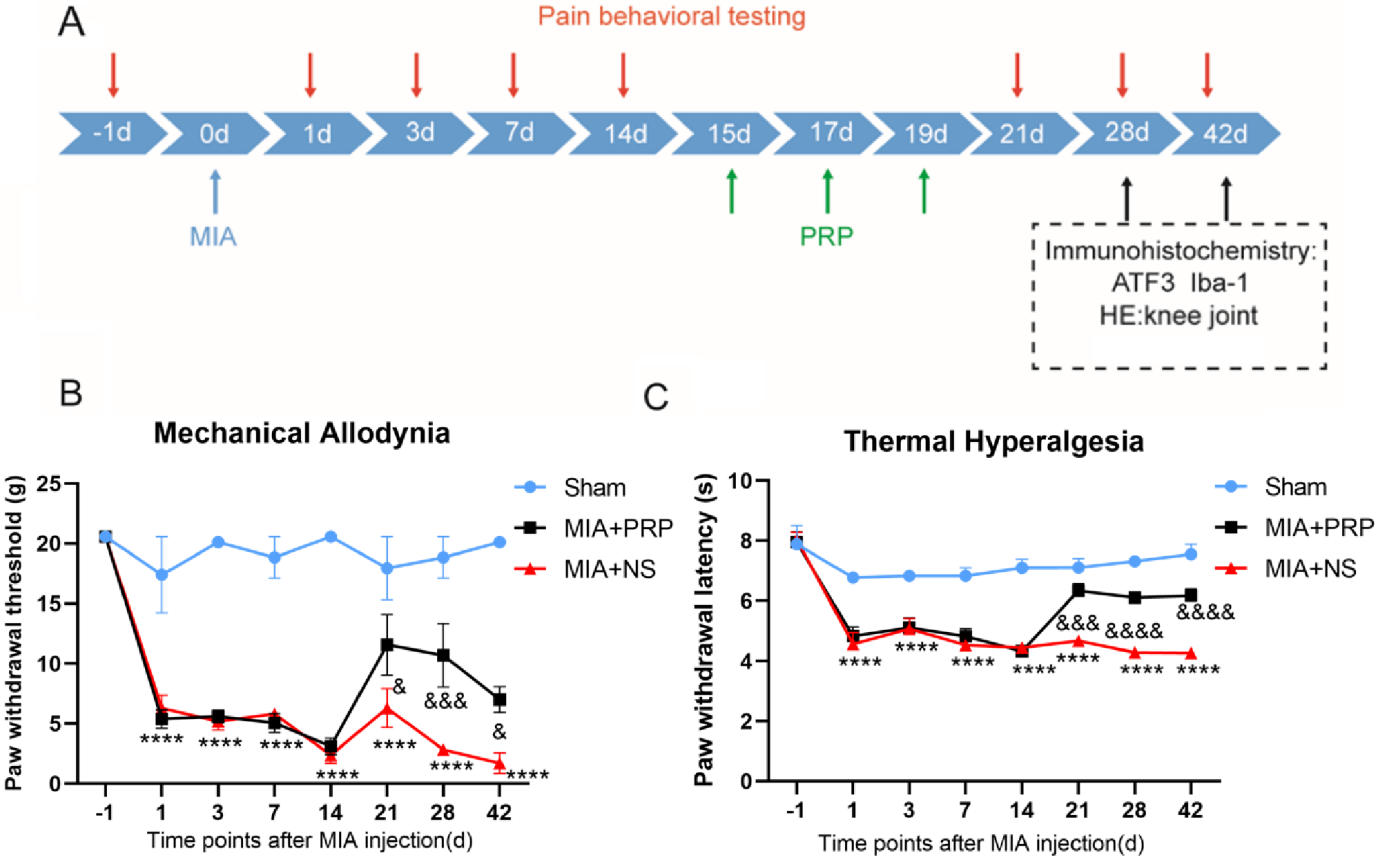
(**A**) Experimental design. (**B** and **C**) Effects of PRP on mechanical allodynia (presented by PWT) and thermal hyperalgesia (presented by PWL) in MIA-induced NP rats were shown in the figure. Compared with the sham group, there were significant decreases on PWT and PWL at days 1–14 after MIA injection. After PRP treatment, PWT and PWL dramatically increased compared with the MIA + NS group. Data were presented as the mean ± SEM, (*n* = 5/group). ^****^P < 0.0001 represented comparison of MIA + NS with sham group; ^&^*P* < 0.05, ^&&&^*P* < 0.001, ^&&&&^*P* < 0.0001 represented comparison of MIA + PRP with MIA + NS group

The left knee joint of the MIA + PRP group received three intra-articular injections of 60 µl PRP at the 15, 17 and 19 days after modeling, while the MIA + NS group and the sham group received 60 µl NS injection under the same conditions, all of the above injections were performed after anesthesia. PWT and PWL were measured at 21, 28, and 42 days after modeling, respectively. Moreover, 28 and 42 days after MIA injection, 5% isoflurane gas was inhaled through the animal anesthesia machine, and after deep anesthesia, the rats were euthanized by cervical dislocation, then the corresponding tissues were taken for subsequent experiments (Fig. 1A).

### Behavioral testing

Five rats were randomly selected from each group for behavioral measurement at 1 day before modeling and at 1, 3, 7, 14, 21, 28 and 42 days after modeling. Von Frey filament test was used to detect mechanical pain threshold of rats, and Hargreaves test was used to detect thermal pain threshold of rats. That is, the testers were not clear about the specific intervention measures of the test group. The rats were acclimated for 20 min before any pain behavioral measurements were performed.

### Von Frey filament test for mechanical allodynia

The hind PWT was determined using von Frey filaments (North Coast, USA) according to the up-down method to evaluate the mechanical allodynia [27]. Before the Von Frey fiber behavioral test, rats were placed on a plexiglass mesh platform and allowed to acclimatize to the testing environment for 20 min. A series of Von Frey fibers (0.16, 0.4, 0.6, 1.4, 2.0, 4.0, 6.0, 8.0, 15.0, 26 g), starting with 2.0 g fiber fibers, were vertically stimulated to the left plantar skin of the rat for about 5 s, and the foot contraction response of the rat was recorded (O : If there is a foot shrinking or foot licking reaction, X: no reaction), if there is a foot shrinking reaction, the test is repeated with the adjacent small first-order fiber, and if there is no reaction, the test is repeated with the adjacent large first-order fiber until there is an “OX” or “XO”. According to the above principle, test again 4 times, each test interval of 5 min, and finally get a series of “X” and “O”. The PWT of rats was finally obtained by substituting the corresponding data according to the following formula:

$$50\text{\%} \text{g}\; \text{t}\text{h}\text{r}\text{e}\text{s}\text{h}\text{o}\text{l}\text{d} =\frac{\left(10\left[{X}_{f}+{K}_{\&}\right]\right)}{\text{10,000}}$$

### Hargreaves test for thermal hyperalgesia

Thermal hyperalgesia was determined in rats by measuring PWL in response to a radiant heat source based on a previously described method [28]. The rats were placed in Plexiglas chambers and stabilized for 20 min. A constant heat source (IITC Model 336 Analgesia Meter, CN) was projected onto a portion of the hind paw, and the response time of PWL was measured. The stimulus was stopped automatically after 20 s to avoid paw injury. The time of foot licking and jumping of the rats was recorded for 3 times, and the time between each test was stopped for 5 min. The results of the 3 tests were averaged to calculate the PWL of the rats [29].

### Immunohistochemistry

The rats in the three groups (*n* = 5/group) were deeply anesthetized and sequentially perfused with saline and 4% paraformaldehyde (pH 7.4) at days 28 and 42. Spinal cord segments L3-L5 and DRG at L3-L5 were extracted from rats. The tissues were post-fixed in 4% paraformaldehyde for 2 h at room temperature and then transferred to 20% sucrose solution overnight and stored at 4 °C. Paraffin-embedded sections of DRG and spinal cord were cut at 5 μm thickness and treated with 0.3% Triton X-100, and 3% hydrogen peroxide in phosphate-buffered saline (PBS) for 1 h, processed for 2 h in 5% normal goat serum, and then stained with primary antibodies overnight at room temperature. DRG specimens were processed using a rabbit antibody against ATF3 (1:100; Immunoway, China), and spinal cord specimens were processed using a rabbit antibody against ionized calcium-binding adapter molecule-1 (Iba-1) (1:1000; Abcam, USA). On the second day, the sections were incubated with secondary antibodies (1:1000, Bioss, China) for 1 h after washing with PBS. Next, the DAB color-developing solution was added until a brown-yellow color appeared, and these sections were re-dyed with hematoxylin after flushing with running water for 30 min, dehydrated using a series of ethanol washes, and cleared in xylene. Images were captured using a Nikon normal forward fluorescence microscope.

### Hematoxylin-eosin (HE) staining

To evaluate cartilage quality, sections were stained with HE (*n* = 5/group). The resected limbs were cut at the mid-femur and mid-tibia and immersed in buffered paraformaldehyde fixative at 4 °C for 1 week. The specimens were continuously demineralized in 10% EDTA for two weeks, followed by standard histological processing using paraffin blocks for subsequent coronal (dorsoventral) sectioning. The samples were serially sectioned in steps of 5 μm stained using HE and assessed by light microscopy. Cartilage degeneration of the medial compartment was scored using the Osteoarthritis Research Society International (OARSI) score [30, 31].

### Statistical analysis

All data are expressed as mean ± SEM. in this study and were analyzed using GraphPad Prism 8.0 software (GraphPad Software, CA, USA). Pain thresholds were evaluated using two-way repeated measures analysis of variance (RMANOVA). One-way ANOVA was used to analyze the differences between the groups for immunohistochemistry staining and HE staining. In all cases, p-values < 0.05 were considered statistically significant.

## Result

**1. Effects of MIA and PRP on Mechanical Allodynia and Thermal Hyperalgesia.**

No significant differences were observed in PWT and PWL among all groups before MIA injection. MIA + PRP and MIA + NS groups showed prolonged allodynia and a significant decrease in PWT and PWL compared to the sham group at 1, 3, 7and 14 days after the MIA injection. As previously mentioned, the rats were treated at 15, 17 and 19 days. Behavioral analysis showed that PWT and PWL in MIA + PRP group were higher than those in MIA + NS group at day 21 after modeling, although PWT and PWL in MIA + PRP group and MIA + NS group showed a downward trend, but MIA + PRP group was consistently higher than MIA + NS group at 28 and 42 days after modeling, indicating that PRP has an analgesic effect on NP induced by KOA in rats (Fig. 1B and C).

**2. Effects of PRP on the protein expression of ATF3 in the DRG and Iba-1 in Spinal Dorsal Horn (SDH)**.

**Fig. 2:**
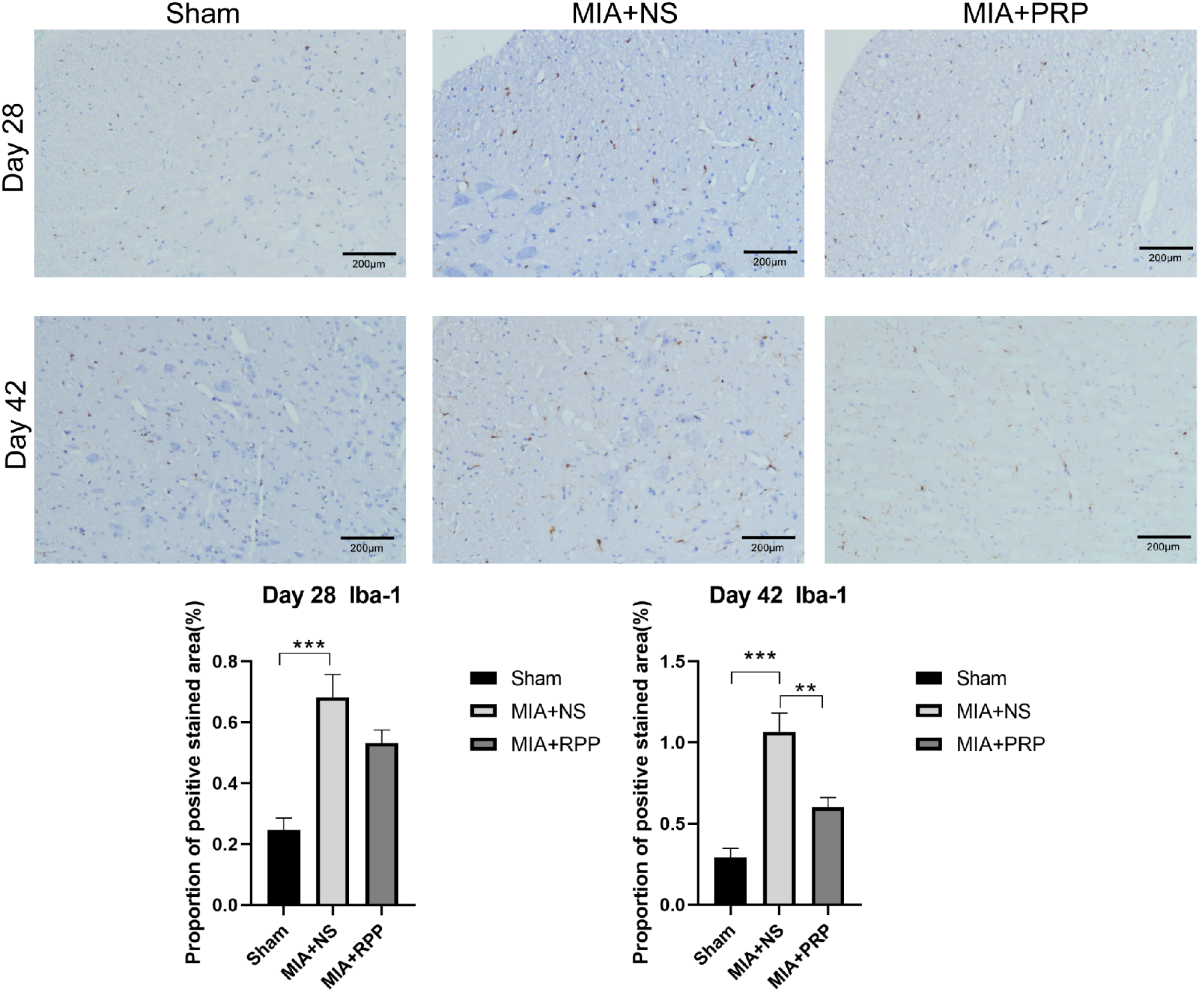
Immunohistochemical staining for Iba-1 in all groups. Scale bars: 200 μm. The proportion of positive stained area were presented as the mean ± SEM (*n* = 5/ group). ^***^P < 0.001, ^**^P < 0.01

**Fig. 3:**
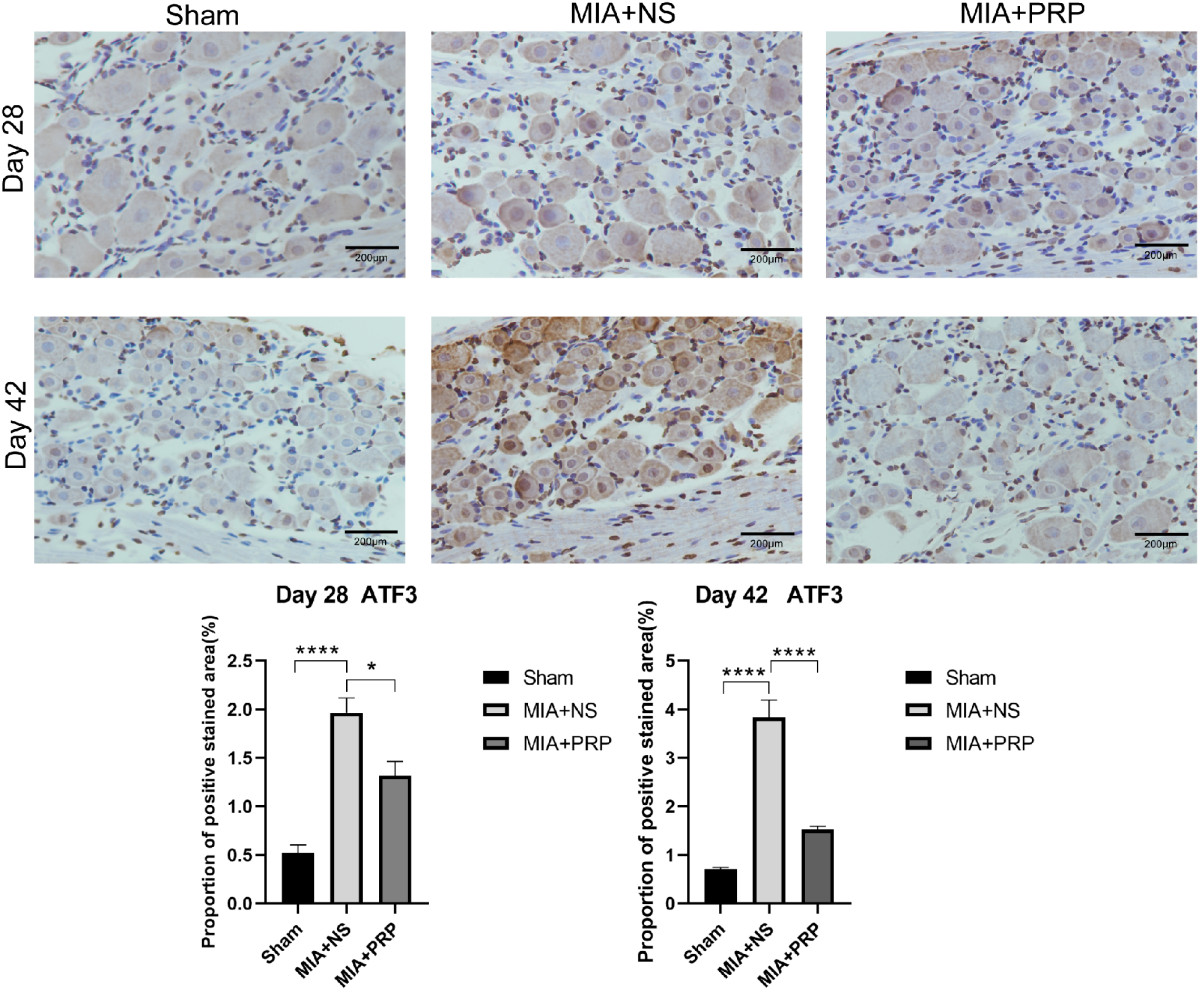
Immunohistochemical staining for ATF3 in all groups. Scale bars: 200 μm. The proportion of positive stained area were presented as the mean ± SEM (*n* = 5/ group). ^*^P < 0.05, ^****^P < 0.0001

The expression of Iba-1 in the SDH and ATF-3 protein in the DRG of rats in each group were observed using immunohistochemical staining at 28 and 42 days after modeling. Compared with MIA + NS group, there was no significant difference in the expression of Iba-1 in SDH of MIA + PRP group at 28 day, and that was lower than that of MIA + NS group at 42 day after modeling (*P* < 0.01) (Fig. 2).Similarly, at 28 days after modeling, the expression of ATF3 started decreasing compared with that in the MIA + NS group (*P* < 0.05), and the effects lasted for 42 days after modeling(*P* < 0.0001) (Fig. 3).

**3. Effect of PRP on Progression of Cartilage Degeneration.**

**Fig. 4:**
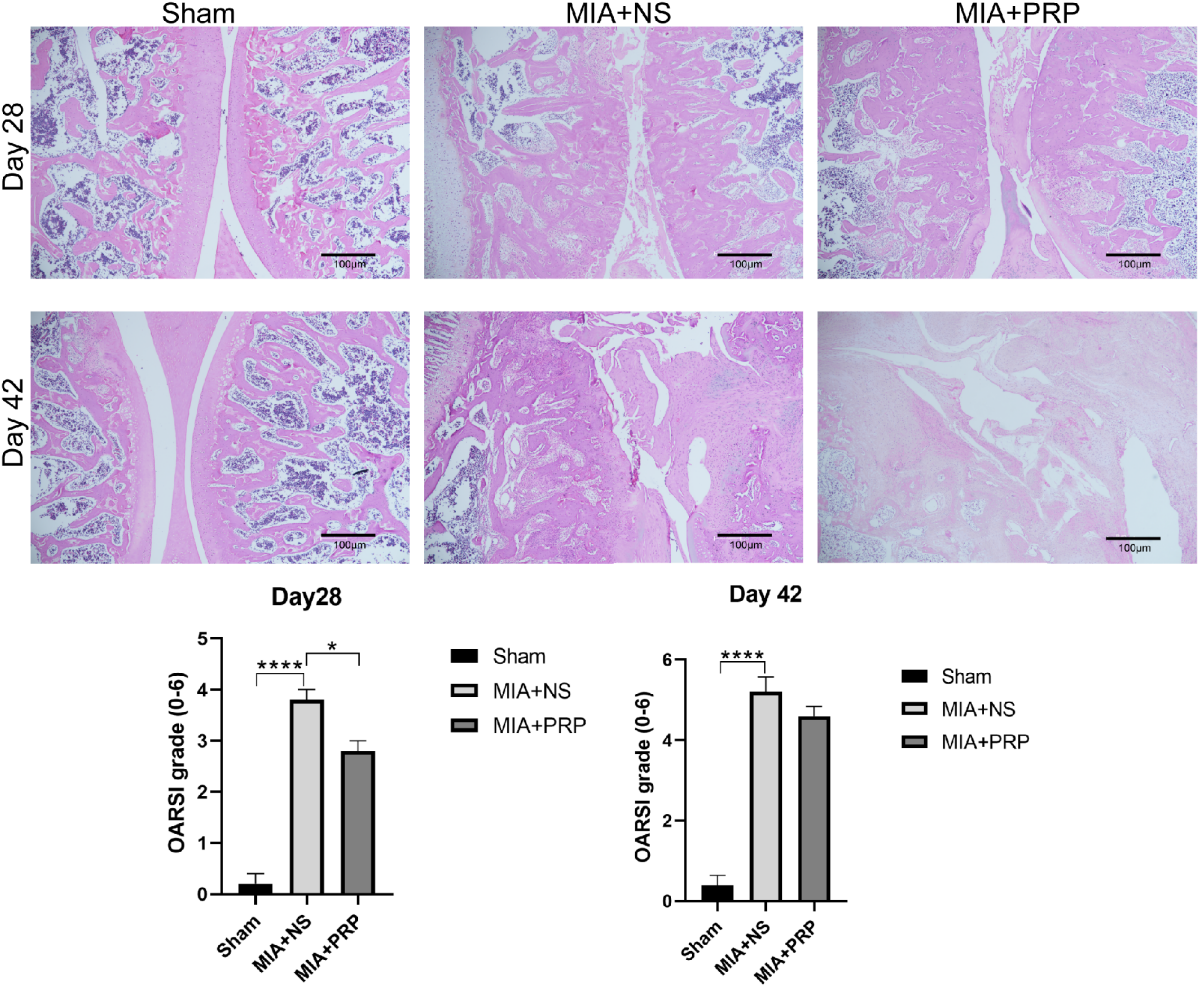
Histological analyses for cartilage. OARSI score was used to assess the histological features of KOA. Scale bars: 200 μm. Representative histological features of the knee joint are shown in the figure. OARSI histological scores for knee were presented as the mean ± SEM (*n* = 5/ group). ^*^P < 0.05, ^****^P < 0.0001

The OARSI scores were used to quantify the histological features of KOA. OARSI scores in MIA + NS group were higher than that in sham group at day 28 and day 42(*P* < 0.0001), and higher than that in MIA + PRP group at day 28 (*P* < 0.05) but not day 42 (Fig. 4).

## Discussion

KOA, especially in the late stage, can be accompanied by nerve injury and NP symptoms, including spontaneous pain and hyperalgesia [32]. Traditional treatments are limited in efficacy and inevitably lead to complications. Nowadays, PRP is emerging as a novel therapy in KOA therapy, due to its preferable clinical effects and high safety [21]. However, to date, there is a lack of evidences of the analgesic effects and potential mechanisms of PRP on NP induced by KOA. Our research firstly reported injection of PRP could relieve NP induced by KOA and reveal the underlying mechanisms.

In our study, a stable NP model was developed by injecting 4.8 mg of MIA into the knee joint for two weeks. We measured symptoms of arthritic pain, including hyperalgesia, allodynia [33] using the Von Frey filament test and Hargreaves test [34]. The results of our behavioral test showed that PWT and PWL began to decrease at 1 day after MIA injection, and then continued to decrease until 14 days after injection. The results proved that high doses of MIA intra-articular injection could induce persistent mechanical hyperalgesia and thermal hyperalgesia, indicating that NP was dominant in this KOA pain model. This is consistent with the results of previous studies that injection of high dose MIA (≥ 2.0 mg/ joint) into the knee joint could cause irreversible structural changes and persistent pain. Sustained inflammatory stimulation and exposure of subchondral bone nerve endings led to injury of primary articular afferent nerve, resulting in NP [32, 35]. Moreover, ATF3 expression in DRG in MIA + NS group was significantly higher than that in sham group at days 28 and 42 after modeling (*P* < 0.0001). ATF3 was a selective marker of nerve injury [37], suggesting that high doses of MIA could induce NP. In summary, our results provided further insights of neural damage in this model.

We adopted the method of multiple injection and chose three doses of intra-articular PRP injections to rats at 15, 17 and 19 days after modeling according to previous studies [36]. Using pain behavioral tests, we found that mechanical and thermal pain thresholds were significantly higher in the MIA + PRP group than in the MIA + NS group at day 21(*P* < 0.05, *P* < 0.001), and this effect lasted until 42 days after modeling (*P* < 0.05, *P* < 0.0001). The results suggested that PRP injection has an analgesic effect on NP induced by KOA.

Microglia are resident immune cells of the central nervous system [16, 37, 38] which are activated in the dorsal horn of the spinal cord following peripheral inflammation and neuropathy. Microglia activation plays an important role in the occurrence and maintenance of chronic NP [17]. Injury of peripheral sensory afferent nerve leads to abnormal excitability of neurons, inducing degeneration of nerve fibers and change of channel expression [13]. Then the microglia in dorsal horn of spinal cord is activated, manifested as proliferation and morphological changes of microglia, and the expression of its marker Iba-1 is upregulated. Previous studies have shown that the number of activated microglia is positively correlated with enhanced joint pain behavior at 7 days after MIA injection [17]. Other studies have shown that intrathecal injection of microglial inhibitors (such as minocycline) can reduce neuroinflammation and NP by inhibiting microglial function. The above studies have confirmed that activation of microglia in spinal cord can lead to persistent pain after tissue trauma or nerve injury [39], and inhibition of microglia activation can alleviate pain. In our study, there was no significant difference in the expression of Iba-1 in SDH between MIA + PRP group and MIA + NS group (*P* > 0.05) at 28 days after modeling. Further observation at day 42, the expression of Iba-1 in SDH in MIA + PRP group was significantly reduced compared with control group (*P* < 0.01). The results indicated that the injection of PRP in knee joint could gradually inhibit the activation of spinal microglia over time, and then alleviated the NP caused by KOA. Therefore, we speculated that the knee joint injection of PRP could inhibit the activation of spinal microglia and achieve analgesic effects.

Multiple mechanisms are known to enhance microglial reactivity in chronic NP, especially peripheral nerve injury. Peripheral sensory nerve injury causes abnormal excitability of nerve endings, induces degeneration of nerve fibers and changes in channel expression (such as significantly increased expression of ATP in the spinal cord) [13], and then promotes spinal microglia activation mediated by P2 × 4 and P2 × 7 receptors [14, 15]. Previous studies found that reducing ATF3 expression in DRG alleviated NP caused by sciatic nerve ligation in rats [40]. These results confirmed that reducing peripheral nerve injury was conducive to alleviating NP. To evaluate afferent nerve injury, we observed ATF3 expression in the DRG by immunohistochemical staining. Compared with MIA + NS group, ATF3 expression decreased in MIA + PRP group at day 28 (*P* < 0.05), and this effect lasted until 42 days after modeling (*P* < 0.0001). From the perspective of time, the downregulation of ATF3 expression in the DRG in MIA + PRP group was earlier than the downregulation of Iba-1 in the spinal cord than that in MIA + NS group. Therefore, we hypothesized that PRP likely downregulated microglial activation by alleviating nerve damage in the sensory system, and ultimately alleviating NP.

HE staining was performed on the left knee joint of rats at days 28 and 42 then OARSI score was performed to assess degree of cartilage injury. It was found that the OARSI score of MIA + NS group was higher than that of MIA + PRP group at day 28 (*P* < 0.05),and there was no statistically significant difference between MIA + NS group and MIA + PRP group at day 42, indicating that in the KOA model induced by high dose MIA, PRP injection still had a certain repair effect on KOA cartilage injury, but failed to reverse cartilage degradation, which was consistent with other study [41].In addition, the pain was still relieved at day 42, which may be due to the absence of nerve distribution and pain fibers in the cartilage itself. Knee pain is more closely related to myelopathy, synovitis and knee effusion [42, 43]. However, the observation time of this experiment was merely at days 28 and 42, and the effect of PRP injection on cartilage repair and its association with pain relief remained to be further observed.

## Limitation

This experiment only preliminarily confirmed that PRP could reduce NP induced by KOA, further investigation was needed to clarify the detailed mechanisms for relieving pain. In subsequent studies, the mechanical sensitivity of the saphenous nerve, that is, the afferent fibers of the joint, can be measured using the joint afferent electrophysiology and vivo calcium imaging technique to determine whether PRP reduces the excitability of nociceptors associated with joint injury. In addition, due to the inability of animal models to fully replicate all characteristics of human KOA, it is imperative to further validate the analgesic effects of PRP on NP induced by KOA in humans through meticulously designed clinical trials.

## Conclusion

In summary, intra-articular injection of PRP may be a potential therapeutic method to NP induced by KOA, and its analgesic mechanism may be related to inhibiting microglia activation and alleviating nerve injury. This study offers a better understanding of NP induced by KOA pathophysiology and further development for future research in the field of PRP treatment of NP.

## Data Availability

The datasets used during the current study available from the corresponding author on reasonable request.

## Abbreviations

KOA: Knee osteoarthritis
NP: Neuropathic pain
MIA: Monosodium iodoacetate
PRP: Platelet-rich plasma
NS: Normal saline
SD: Sprague–Dawley
PWT: Paw withdrawal threshold
PWL: Paw withdrawal latency
HE: Hematoxylin-eosin
DRG: Dorsal root ganglia
SDH: Spinal dorsal horn
PBS: Phosphate-buffered saline
Iba-1: Ionized-calcium-binding adapter molecule-1
ATF3: Activating transcription factor 3
RMANOVA: Repeated measures analysis of variance
OARSI: Osteoarthritis Research Society International
ATP: Adenosine triphosphate
